# Delivering vaccines for the prevention of pneumonia — programmatic and financial issues

**DOI:** 10.15172/pneu.2013.2/244

**Published:** 2013-02-14

**Authors:** Diana C. Otczyk, Allan W. Cripps

**Affiliations:** School of Medicine, Griffith Health Institute, Griffith University, Gold Coast Campus, Queensland, 4222 Australia

**Keywords:** pneumonia, bacteria, *Haemophilus influenzae* type b, *Streptococcus pneumoniae*, vaccine, Global Action Plan, GAPP, GAVI, WHO, pneumonia.org.au, World Pneumonia Day

## Abstract

Pneumonia is the leading cause of morbidity and mortality in children younger than 5 years. Vaccines are available against the main bacterial pathogens *Haemophilus influenzae* type b and *Streptococcus pneumoniae*. There are also vaccines against measles and pertussis; diseases that can predispose a child to pneumonia. Partners such as the Global Alliance for Vaccines and Immunisation (GAVI), the Hib Initiative, the Accelerated Development and Introduction Plan for pneumococcal vaccines and the Measles Initiative, have accelerated the introduction of vaccines into developing countries. Whilst significant improvements in vaccine coverage have occurred globally over the past decade, there still remains an urgent need to scale-up key pneumonia protection and treatment interventions as identified in the Global Action Plan for the Prevention and Control of Pneumonia (GAPP). There is promise that global immunisation will continue to improve child survival. However, there are several challenges to vaccine implementation that must first be addressed, including: a lack of access to under-served and marginalised populations; inadequate planning and management; a lack of political commitment; weak monitoring and surveillance programmes and assured sustainable finance and supply of quality vaccines. There is an urgent need to increase global awareness of the devastation that pneumonia brings to the worlds poorest communities.

## 1. Introduction

Pneumonia is responsible for 18% of the estimated 8.8 million child deaths [1] — more than AIDS, malaria and measles combined — representing a global burden of over 4,000 children dying every day. Pneumonia is a disease that occurs worldwide; however, the burden of disease occurs predominantly in impoverished communities that lack access to basic healthcare including, antibiotics and oxygen therapy [2]. Ten African and Asian countries have the most child pneumonia deaths — 50% of deaths occur in five countries [3]. Compared to a child in a developed country, a child in a developing country is 17 times more likely to die before reaching the age of 5 [1] with a 215 times greater chance of dying from pneumonia [4].

A significant proportion of serious childhood invasive disease and pneumonia morbidity and mortality are attributed to the pathogens *Streptococcus pneumoniae* (pneumococcus) [5] and *Haemophilus influenzae* type b (Hib) [6]. Recognising the significant impact that vaccines might have on morbidity and mortality caused by pneumonia, the World Health Organization (WHO) recommended in November 2006 and March 2007 that Hib and pneumococcal conjugate vaccines, respectively, be routinely used in the immunisation schedules of all countries [7, 8]. The currently licensed pneumococcal and Hib conjugate vaccines have been extensively reviewed elsewhere [9]. There are three pneumococcal conjugate vaccines: the 7-valent vaccine (PCV-7), the 10-valent vaccine (PHiD-CV) and the 13 valent vaccine (PCV-13). The Hib conjugate vaccine may be administered separately or combined with one or more of the following antigens: diphtheria, tetanus, pertussis (DTP), hepatitis B (HepB) and/or inactivated oral polio vaccine (OPV). WHO recommends routine infant immunisation schedules include vaccination with Hib in the combined DTP/HepB pentavalent vaccine. Whilst the vaccines have experienced rapid uptake in industrialised countries, Hib conjugate vaccines have been underutilised in high burden countries and the introduction of the pneumococcal conjugate vaccines has been slow. Despite this slow uptake, it may be argued that these vaccines have been introduced into some countries without adequate monitoring of vaccine effectiveness.

The United Nations fourth Millennium Development Goal (MDG4) has as its target a two-thirds reduction in the under-five mortality rate from its 1990 level by 2015 [10], however, over the last 20 years there has been a lack of substantial progress towards mortality reduction with only a 28% decline in child deaths since 1990, far short of its 67% target [11, 12]. Because of its significant impact on child mortality, pneumonia stands as the greatest threat behind global efforts to achieving the MDG4. Two initiatives have laid the framework for accelerating the progress to tackle childhood pneumonia. The Global Action Plan for Prevention and Control of Pneumonia (GAPP) arose following the ‘ISPPD Declaration’ at the Fifth International Symposium on Pneumococci and Pneumococcal Diseases held in Alice Springs, Australia, in 2006 [13], as an initiative to accelerate the utilisation of proven interventions for the prevention and control of pneumonia. The second initiative was the World Health Assembly’s endorsement of GAPP in 2010 and its resolution that all countries should establish evidence-based national policies and action plans to strengthen health systems and to monitor progress against childhood pneumonia [14].

## 2. Delivering vaccines to developing countries

### 2.1. Global partners in immunisation

The Global Alliance for Vaccines and Immunisation (GAVI) Alliance was established in 2000 in an effort to stop the inequity in vaccine access between developing and industrialised countries. GAVI and its partners, WHO, United Nations International Children’s Emergency Fund (UNICEF), the Program for Applied Technology in Health (PATH), the US Centres for Disease Control (CDC), the Independent Vaccine Initiative (IVI), the World Bank, Bill & Melinda Gates Foundation, the Sabin Vaccine Institute and its Pneumococcal Awareness Council of Experts (PACE) and the Hib Initiative project have all been working to accelerate the uptake of vaccines in developing countries.

In 2009, GAVI, the World Bank, WHO, UNICEF, the governments of Italy, UK, Canada, Russia and Norway and the Bill & Melinda Gates Foundation launched the Pneumo Advanced Market Commitment (AMC) committing $1.5 billion to accelerate the development, production and introduction of affordable pneumococcal vaccines for developing countries. In 2010, GSK and Pfizer Inc. entered 10-year commitments with UNICEF to supply 30 million doses per annum of PHiD-CV and PCV-13, respectively, at a greatly reduced price compared to industrialised countries. Further support has been forthcoming via the International Finance Facility for Immunisation which had raised US$ 1.9 billion, via investments in the capital markets, to be channelled by GAVI towards investments in immunisation initiatives in developing countries [15].

As of September 2012, 179 countries have introduced Hib/pentavalent vaccines in infant immunisation schedules [16]. Of these, Russian Federation, Belarus, India and Nigeria have partially introduced the vaccine. A further 10 countries plan to introduce the vaccine during 2012 to 2013, including Haiti, Japan, Maldives, Myanmar, Singapore and Timor-Leste, Egypt, Indonesia, Iran and Somalia. A total of 78 countries are using pneumococcal conjugate vaccines in their national immunisation programs [17]. Since the first pneumococcal conjugate vaccine was introduced in Rwanda, in 2009, a further 14 countries in Africa have taken up the vaccine, including Benin, Botswana, Burundi, Cameroon, Central African Republic, Democratic Republic of the Congo, Ethiopia, Gambia, Ghana, Kenya, Malawi, Mali, Sierra Leone and South Africa. Globally, an additional 15 countries are scheduled to introduce the vaccine in 2012 and a further 24 in 2013.

### 2.2. New emerging markets

In 2010, the estimated worth of pharmaceutical markets in developing countries with emerging economies (Brazil, Russia, China, India, Turkey, Mexico, and Indonesia) was estimated at around US$ 136 billion [18]. Vaccine research and development in India and China is emerging as one of the growth drivers with India reportedly producing 60% of the global vaccines [19]. Major pharmaceutical companies have been motivated to invest in and partner with developing countries due to an emerging favourable and predictable economic environment, facilitated by the increased purchases of new vaccines, and the implementation of supportive regulatory strategies and intellectual property protection. Under a WHO prequalification system, UN procuring agencies are able to purchase vaccines of assured quality, safety and efficacy for the provision of national immunisation schemes. Currently, of 10 manufacturers that have Hib/Hib containing vaccines on the prequalified list, 6 are from emerging manufacturers [20]. The Trade-Related Aspects of Intellectual Property Rights (TRIPS) Agreement [21] requires World Trade Organization members to establish minimum standards for protecting and establishing intellectual property rights, globally. The TRIP’s Agreement also addresses specific actions for relevant stakeholders which are designed to promote-health related innovation, especially to meet the research and development needs of developing countries, and to promote technology transfer.

## 3. Opportunities and challenges

In the last decade there have been unprecedented opportunities for the prevention and treatment of pneumonia with an influx of resources and investment from organisations. Yet, millions of children continue to die unnecessarily from this deadly disease. Challenges remain in progressing the interventions identified by GAPP, including, risk reduction through good nutrition, exclusive breastfeeding and limiting exposure to indoor smoke, as well as vaccination and effective case management. Two thirds of childhood deaths due to pneumonia can be avoided if these proven interventions were scaled up to 90% coverage and were focused on the most marginalised children [22]. Proven vaccines against pneumococcus, Hib, measles and pertussis are available and are recognised as safe and effective tools for the prevention of childhood pneumonia. In the ‘Decade of Vaccines’ [2011–20], childhood immunisation rates of 6 key vaccines, including measles, DTP, pneumococcal and Hib conjugate vaccines, will be increased. This could help prevent up to 2.7 million child deaths from pneumonia [23].

### 3.1. Vaccination against thefour leading causes of pneumonia

Coverage of vaccines such as measles and DPT have been expanded as a result of a number of strategies, including: expanded routine services to reach-out into communities; eradication programs such as the Measles Initiative and the Reaching Every District strategy in Africa and Asia [24–26]. In 2010, an estimated 85% of the birth cohort or 109.4 million children aged < 12 months were immunised with 3 doses of DTP3, a 5% increase in the estimated coverage in 2006 (80%) [27]. Whilst this outcome is promising, there are a number of countries in Africa and South-East Asia where coverage will only be in the order of 60–70%. Of the approximate 19.3 million children who did not receive DTP3 during their first year of life, three countries — India, Nigeria and the Democratic Republic of Congo accounted for half of the children. Since 2006, there has been a 4% increase in coverage with the measles vaccine to the present day 85%, however, again in Africa and South-East Asia coverage is estimated to be 76% and 79%, respectively [27]. Because of the decline in deaths, there is a real concern that measles may be viewed by governments and the global health community as no longer being a threat and erode the gains that have been made over the last 5 years. Since 2009, there have been widespread measles outbreaks affecting 30 countries in sub-Saharan Africa and India [28].

GAVI, the Hib Initiative [29], the Accelerated Development and Introduction Plan for pneumococcal vaccines (PneumoADIP) and the Pneumo Ad-hoc introduction group have worked with countries to facilitate the introduction of Hib and pneumococcal conjugate vaccines, respectively. While the majority of countries are providing the Hib-containing vaccine in their infant immunisation schedules, less than 50% of children worldwide are fully vaccinated with 3 doses of the vaccine (Hib3) [16]. Substantial variations in Hib3 vaccination coverage among South East Asian countries and in Africa are still seen. For example, the average Hib3 coverage for Africa is 62% while the country of Chad has achieved only 6% coverage [1]. Despite the fact that most of infant morbidity and mortality due to pneumonia occur in Africa and South East Asia, few countries have introduced pneumococcal and/or Hib conjugate vaccines into their immunisation schedules — including countries with large birth cohorts such as India, China and Indonesia [16, 17]. There is hope that this situation may improve, albeit slowly, in the near future with Bangladesh indicating that it intends to take-up the pneumococcal vaccine in 2013 and the Indian government approving the phased introduction of the Hib conjugate vaccine. The Indian states of Kerala and Tamil Nadu introduced the Hib conjugate vaccine in their immunisation program late 2011 [30].

### 3.2. Advocating for immunisation as a cost-effective health intervention

The availability of the Hib and the new pneumococcal conjugate vaccines has prompted national policymakers to assess the projected health benefits, cost and cost-effectiveness of vaccination when considering their inclusion in national immunisation programmes. Several reviews of economic evaluation studies have concluded that the vaccines are relatively cost-effective interventions [31–33]. However, it was also noted that only a few studies were from resource poor countries and these were of poor quality.

As the Hib and pneumococcal burden is difficult to establish in low resourced countries and since Hib and the new pneumococcal conjugate vaccines remain relatively expensive, there is uncertainty about their economic value to a country and reluctance on the part of policy-makers to include them in national immunisation schedules. In recent years, there has been an expansion in international expertise and support available to these countries in the form of technical bodies and programmes such as the National Immunisation Technical Advisory Groups (NITAGs) and the Pan-American Health Organization (PAHO) ProVac Initiative in the Americas [34]. Today, 114 countries claim the existence of a NITAG, however, less than half of these countries meet the required established standards [35]. While these entities can assist policy-makers in making evidence-based decisions regarding new vaccine introduction, their effectiveness may be undermined by the quality and quantity of epidemiological data available. Uncertainty about the burden of disease data in resource-poor countries may necessitate generalisations to be made or data may be extrapolated from countries with different demographics [36, 37] whilst temporal changes in pneumococcal serotype epidemiology can impact on herd immunity and the cost-effectiveness of a vaccine program [38–40]. Furthermore, a lack of validation and transparency of cost-effectiveness models may limit their intended usefulness [41] producing differing results regarding the cost-effectiveness of Hib and pneumococcal conjugate vaccines and their impact on morbidity and mortality [42].

### 3.3. Strengthening the capacity of integrated health systems

The underlying weakness of health systems in many developing countries can have a significant negative influence on immunisation coverage and the treatment of pneumonia. As infants are not fully immunised until 6 months of age, community case management strategies such as the ‘Integrated Management of Childhood Illness’ (IMCI) have the potential to significantly reduce mortality from pneumonia [43], however, access to a health care facility for the provision of antibiotics and oxygen therapy is currently insufficient in many countries. Limited by an already weak health system, the implementation of IMCI has been plagued by shortages of financial, material and human resources, lack of support from international agencies and poor working conditions with high turnover of health workers and highly skilled personnel [44–47]. Less than half (42%) of the 68 countries that together account for at least 95% of maternal and child deaths worldwide have adopted a policy at the community level for the administration of antibiotics to manage severe pneumonia [47]. Currently, children in Africa and Asia are still not receiving the correct treatments: only 48% of children with pneumonia are reportedly taken to a qualified medical care provider and 27% are not receiving antibiotic treatment [47]. Progress in reducing neonatal mortality has been slow or non-existent; accounting for 41% of deaths in children younger than 5 years [47] with more than half dying in the first 28 days in southern Asia [48]. Exclusive and early breastfeeding in the first 6 months of an infant’s life has a significant protective effect against pneumonia and other diseases [49], however, only 58% of mothers are initiating early breastfeeding and only 35% are practicing exclusive breastfeeding [47]. Further advocacy is required about the benefits of exclusive breastfeeding.

Poor program and vaccine management systems at the country level make the scale-up of vaccine delivery difficult in many countries resulting in poor vaccine stock management, handling and storage and high wastage. Today, health services are not only faced with the challenges of cold-chain management of vaccines but also managing vaccines that are much more expensive and more difficult to handle in terms of increased volume and increased complexity. In Africa, new innovative partnerships are being explored to leverage the strengths of the private sector to improve vaccine distribution [50] and supply chain management [51]. However, the outsourcing of supply chain management has proved not to be a panacea. It has been a mixed experience in the Western Cape of South Africa with regard to PCV-7 and DTP/OPV/Hib vaccines being introduced into the national immunisation schedule [51]. Experience has shown that this too can bring on a new host of challenges and success is highly dependent on a strong framework already being in place at the local level.

Inadequate surveillance and monitoring systems to assess the impact of Hib and pneumococcal conjugate vaccines on disease patterns are an ongoing concern [52, 53]. Perhaps the greatest threat facing pneumococcal vaccine effectiveness is serotype replacement. The observed increase in serotype replacement in invasive disease following pneumococcal vaccination in industrialised countries has highlighted the importance of robust surveillance systems [54] and warrants particular consideration in developing countries where carriage and invasive disease incidence rates are much higher and resources are limited. The systematic implementation of carriage studies to assess the impact on serotype distribution is urgently needed [54, 55]; WHO recommending surveillance commence at a minimum of 2 years prior to vaccine introduction and continuing 5 years post-introduction [56]. Vaccination schedules for the conjugate vaccines differ between countries and the choice of primary dosing schedule and the need for booster doses continue to be a source of review [57–61]. Following a recent surveillance study in four South American countries with high coverage of Hib conjugate vaccine, PAHO found that the low incidence of Hib invasive disease and nasopharyngeal carriage was sustained using a 3-dose immunisation with or without a booster dose [62]. Accumulating evidence also supports a 2p+1 dose schedule for reducing Hib disease in developing countries [57]. Systematic reviews of data from randomised controlled trials and observational studies using PCV-7, PHiD-CV, PCV-13 and investigational pneumococcal conjugate vaccines PCV-9 and PCV-11 support the use of 2 schedules: a 6, 10, 14 week series or a 2, 4, 6 month series (3p+0) plus a booster at 1–2 years of age (3p+1) [59, 60]. Alternatively, a 2p+1 dose schedule has been recommended [61, 63]. Recently, WHO recommended in their pneumococcal position statement the use of a 2p+1 or 3p+0 dosing schedule [56]. A reduced vaccination schedule may offer a potential financial benefit for a developing country. However, the long-term implications of these dosing variations on trends in disease incidence will require ongoing surveillance, in particular, where protection may be reduced with a 2p+1 schedule in countries that have an increased prevalence of pneumococcal serotype 23F and 6B [58]. In settings where there is high vaccine coverage, such as in the Australian Indigenous population, high rates of Hib disease and Hib carriage [64] poses a continuous threat to invasive disease resurgence if not monitored vigilantly.

### 3.4. Vaccine financing and financial policy

The high cost of the conjugate vaccines introduced into the routine immunisation programs present substantial financial burdens in many low-income countries [65]. At present until 2015, GAVI is committed to enabling eligible countries to purchase the Hib and pneumococcal conjugate vaccines for a small co-payment of 15–30 cents per dose. GAVI pays for the remainder of the vaccine cost, or the tail price. Two factors which may have a significant impact on reducing the price of these vaccines include the increase in the number of emerging manufacturers [66] and the more recent initiative, the Gates Foundation funded Vaccine Product, Price, and Procurement (V3P) project [67]. Following the arrival of emerging manufacturers, the price of the pentavalent vaccine has fallen from US$ 3.75 in 2007 to a low of US$ 2.25 per dose in 2011 [68]. However, the price reduction of the vaccine is still less than expected and would need to come down further for GAVI to be able to sustain long-term funding of the vaccine. To support sustainable vaccine introduction into countries, the V3P project aims to provide policymakers with the necessary data on vaccines to facilitate national decision-making. UNICEF’s decision to increase pricing transparency has revealed wide disparities in what manufacturers are charging for their vaccines. Whilst the intention of this move was to stimulate a more competitive market and lower prices for the newer more expensive pneumococcal conjugate vaccines, it may also push other buyers of vaccines to ask for the lowest prices they can get. Under the arrangements through the AMC, GAVI and the developing countries that introduce the vaccines are able to purchase the pneumococcal conjugate vaccines at the predetermined price of US $3.50 per dose. Pfizer Inc. and GSK receive $3.50 a dose from UNICEF with an additional $3.50 paid by AMC donor financed funds. Even if a competitor made the vaccines for less than the $3.50, Pfizer Inc. and GSK would get subsidies from AMC donor funds to bring the price to $7 per dose. The impact of the pneumococcal AMC and its efficiency and cost-effectiveness are yet to be evaluated. As yet, no further AMCs have been announced.

Strong government commitment is critical in order for health systems to move forward to introduce new vaccines into national immunisation services [69]. The Hib and pneumococcal conjugate vaccines are much more expensive than traditional vaccines such as the measles vaccine which is currently US$ 0.19 a dose [68]. As such, they are now a significant cost component of national immunisation expenditures. In South Africa, for example, with the introduction of three new vaccines, PCV-7, DTP/OPV/Hib and rotavirus, the vaccine cost per fully immunised child increased from US $25 in 2008 to US $175 in 2010 [51]. Compounding this dilemma is GAVI’s ongoing funding challenges. In 2011, GAVI faced a US$3.7 billion funding gap to finance the rollout of new vaccines to 2015. At a recent GAVI pledging conference, donors committed $4.3 billion, exceeding the target set by GAVI [70]. A portion of the pledges is conditional, including a $90 million contribution by the US which is subject to Congressional approval and $250 million of a $1,000 million commitment made by the Bill & Melinda Gates Foundation which will be retained subject to GAVI raising and matching funds from new donors. Whilst this outcome marks a success for GAVI, GAVI’s ability to finance the procurement of pneumococcal conjugate vaccines and meet accelerating country demand for the vaccines will continue to be a challenge in the coming years as global financial uncertainties foster concerns about the long-term sustainability of donor commitments and country co-financing in developing countries. In an attempt to reduce its own costs, GAVI took steps to tighten the country eligibility criteria for funding [71] resulting in approximately 16 countries, as of March, 2011, no longer being eligible for GAVI support nor having access to the vaccine at GAVI prices [72]. This decision, however, resulted in some major countries, including Indonesia, Cuba and The Congo Republic being excluded from GAVI support and threatened the financial viability of the AMC. Consequently, a decision was made to allow the graduated countries to continue to have access to pneumococcal conjugate vaccines through GAVI and the AMC, provided they financed the purchase of the vaccine in full. While it is anticipated that this move would alleviate GAVI’s financial standing, it assumes that those graduated countries will be prepared to or even capable of self-financing the GAVI tail price. For those countries that have included the vaccine in their immunisation schedules, it will be a challenge for them to maintain vaccination programs without ongoing assistance from GAVI. As such, countries are hesitant to introduce new vaccines. Under the AMC, the tail price of PHiD-CV and PCV-13 will be a determining factor in the decision of graduating and graduated countries to introduce these vaccines. The Sustainable Immunisation Financing Program (SABIN) is presently working with African and Asian countries to increase immunisation budgets.

### 3.5. New investments and partnerships

Through increased investment and new partnerships, there are opportunities to accelerate the accessibility of more affordable and tailored vaccines to meet the needs of target countries. In 2010, for example, GSK successfully negotiated a production technology transfer with Brazilian institution, Fiocruz, to develop and produce key paediatric vaccines against diseases affecting Brazil’s population, including Hib and pneumococcal disease [73]. Whilst the TRIPS Agreement offers provisions for technology transfer from multinational pharmaceutical companies to emerging suppliers, for a pharmaceutical company to invest in and partner with a developing country, there must also be a supportive business, regulatory and scientific environment. The transfer of key technologies involved in vaccine manufacture requires that the recipient country has a robust intellectual-property system that is properly managed and enforced. Pharmaceutical companies are also faced with significant challenges to production technology transfer to developing countries. The complexities of manufacturing vaccines with multiple components and stringent quality processes require a degree of scientific and regulatory expertise; all contributing to the cost of and time needed for vaccine production. A point in case is the removal from WHO’s prequalification list of Shan5 (DTwP/HepB/Hib, Shantha Biotech, India) in 2010 [74] and more recently, Easyfive™ (DTwP/HepB/Hib; Panacea Biotechnics, India) [75] following the emergence of quality and safety manufacturing concerns. Shantha Biotech is a subsidiary company of the global Sanofi group; its long-term contract with WHO for the procurement of Shan5 was reportedly worth $340 million [74].

## 4. Conclusion

Substantial gains have been made in improving the overall burden of childhood pneumonia, however, it is apparent that efforts must be focussed on the available pneumonia interventions if the 90% target set by GAPP is to be met. While Hib and pneumococcal conjugate vaccines hold the promise to saving millions of children’s lives, there are the ongoing challenges of reducing the barriers towards vaccine implementation. Countries must take responsibility for establishing strong governance and provide quality immunisation for their people. There should be strong health systems to ensure equitable access to and coverage of immunisation. To allow policy-makers to make informed decisions and implementation strategies, regional epidemiological research and disease surveillance must be expanded to address gaps in knowledge and to monitor the impact of immunisation. The financing of vaccines continues to be a challenge proving too costly for health programs in resource-poor countries to afford. Immunisation programmes must be assured of sustainable access to long-term funding and quality vaccine supply.

Global awareness about pneumonia’s enormous impact on children and communities remains far too low. To this end, movements such as GAPP, The Global Coalition Against Child Pneumonia, World Pneumonia Day and the recently launched ejournal **pneumonia**, aim to focus the attention of the global community around a common health agenda and encourage efforts to fight the world’s leading cause of child death.
